# Role of Collagen in the Etiology of Inguinal Hernia Patients: A Case-Control Study

**DOI:** 10.7759/cureus.43479

**Published:** 2023-08-14

**Authors:** Siva Prasanna, Praveen G Sekaran, Ajay Sivakumar, Vimal K Govindan

**Affiliations:** 1 General Surgery, PSG Institute of Medical Sciences and Research, Coimbatore, IND

**Keywords:** transversalis fascia, skin, collagen type iii, collagen type i, inguinal hernia

## Abstract

Introduction

Technical faults are no longer accepted as the sole reason for recurrence following inguinal hernia (InH) repairs. Medical literature has been studied to find any contributing factors and collagen has emerged as a promising marker. Owing to their long half-lives, it has been found to best reflect the process of scarring, which is central to ensuring the formation of a proper fibrous tissue that incorporates the mesh with the abdominal wall.

Methods

Sixty participants were divided into two groups. The case group were patients diagnosed with InH and the control group had patients undergoing abdominal surgeries for indications other than abdominal wall hernias. A 0.5x0.5cm specimen of skin and transversalis fascia were biopsied and subsequently stained to determine the amount of collagen I and III.

Results

Collagen I, collagen III and the ratio of collagen I to III was measured. Collagen I was normal in the skin of both groups but decreased in transversalis fascia of cases. Collagen III was found to be normal in transversalis fascia of both cases and controls, but increased in the skin of cases. Ratio of collagen I to III was decreased in both skin and transversalis fascia of cases. Statistical analysis was carried out using an unpaired t-test, non-parametric Mann-Whitney test, ANOVA and chi-square test.

Conclusions

Our study has reported that in patients with inguinal hernia, collagen III or immature collagen is increased in skin and collagen I or mature collagen is decreased in the transversalis fascia. The ratio of collagen I/III is decreased in both skin and transversalis fascia.

## Introduction

Inguinal hernias (InH) are a highly prevalent surgical condition. Etiology can be attributed to multiple factors though uncertain. Studies have noted a recurrence of about 5-30% despite prior surgical repair [1]. Hence, it becomes important to recognize the exact pathology within. The long-held belief is that weakness of the abdominal wall and a rise in intra-abdominal pressure predispose to hernia formation. However, recent studies have alluded to the role of connective tissue biology [2,3].

If we develop biomarker tests that could reveal contributing factors to hernia development such as disturbances in the collagen profile, or properly map the gene profile for InHs subtypes, then the possibilities for precision-based medicine/surgery would expand. Tissues such as rectus sheath, cremaster, hernial sac, and even skin have been analyzed for such prospects [3,4]. The role of collagen in providing structural integrity to the aponeurosis and fascias has been studied [2,5]. Any collagen aberration presents as a posterior wall weakness and direct hernia [6]. Correspondingly, any aberration in the transversalis fascia leads to the formation of an indirect InH.

Collagen is the most abundant extracellular matrix protein. The ratio and level between collagen type I and type III basically determine the strength of both connective tissue and scar tissue [7]. A study in 2017 showed that individuals with InH display altered connective tissue, compared with controls regarding the ratio of collagen fibers, fascia architecture, and level of enzymes involved in connective tissue homeostasis [8]. Disorders like Marfan's, Ehlers-Danlos, and Hurler-Hunter, polycystic kidney disease, and osteogenesis imperfecta are at increased risk of hernia formation [9]. This point towards a systemic connective tissue abnormality as the cause. They are also found to be inherited among certain family members which may indicate a multifactorial inheritance pattern [10].

There are several debatable reports on the use of collagen as a marker. Some studies have reported no difference in the collagen composition of transversalis fascia while others suggest a significant difference in the rectus sheath predisposing to hernias [2,11]. There are not many studies done in the Indian population and we aim to fill this lacuna. In our study, we utilize immunochemical staining methods to determine the percentage of collagen type I and type III in the skin and transversalis fascia of patients diagnosed with inguinal hernia.

## Materials and methods

A case-control study was performed in our center investigating 60 patients who fulfilled the inclusion and exclusion criteria. All patients provided informed consent for participation. The study adhered to the guidelines set in the Declaration of Helsinki. The study protocol was approved by the Institutional Ethics Committee of our institute (project no. 15/331).

Inclusion criteria for the case group involved patients with a diagnosis of primary inguinal hernia, both direct and indirect. The exclusion criteria included those with diabetes mellitus, hypertension, chronic obstructive pulmonary disease, connective tissue disorder, smoker, recurrent hernias, on steroid medications, < 18 and > 75 years of age, and those who had undergone previous abdominal surgeries. The control group contained patients planned for other laparoscopic procedures in the abdomen such as cholecystectomy, gastrectomy, colectomy, and ovarian cystectomy. 

**Figure 1:**
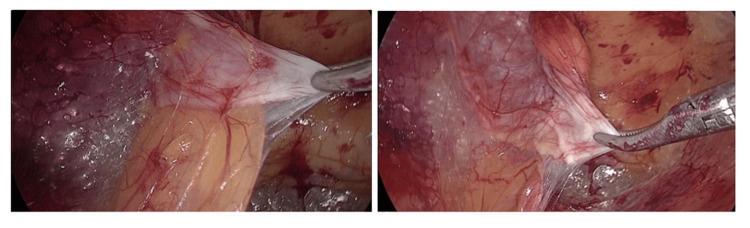
Biopsy from transversalis fascia

All patients were received in the outpatient clinic and informed about the research protocol. Once they provided consent, they were assessed and planned for laparoscopic hernia repair. During surgery, once the diagnosis had been confirmed, a 0.5x0.5 cm size piece of the transversalis fascia was obtained from the inguinal triangle limited by the lateral border of the rectus sheath, inferior epigastric vessels, and the inguinal ligament (Figure 1). A similar 0.5x0.5 cm piece was excised from the skin while closing the skin incision on the anterior abdominal wall. 

These samples were subsequently fixed. Formalin (10%) was used and submitted for histology. Sections of 4 µm were obtained and stained in two groups, one with hematoxylin and eosin (H&E) and the other with Masson’s trichrome to confirm the presence of collagen fibers. Separate slides coated with poly-L-lysine underwent immune-histochemistry staining for the detection of collagen I and III. The adequacy of the material was evaluated based on the sections stained by H&E. 

**Figure 2:**
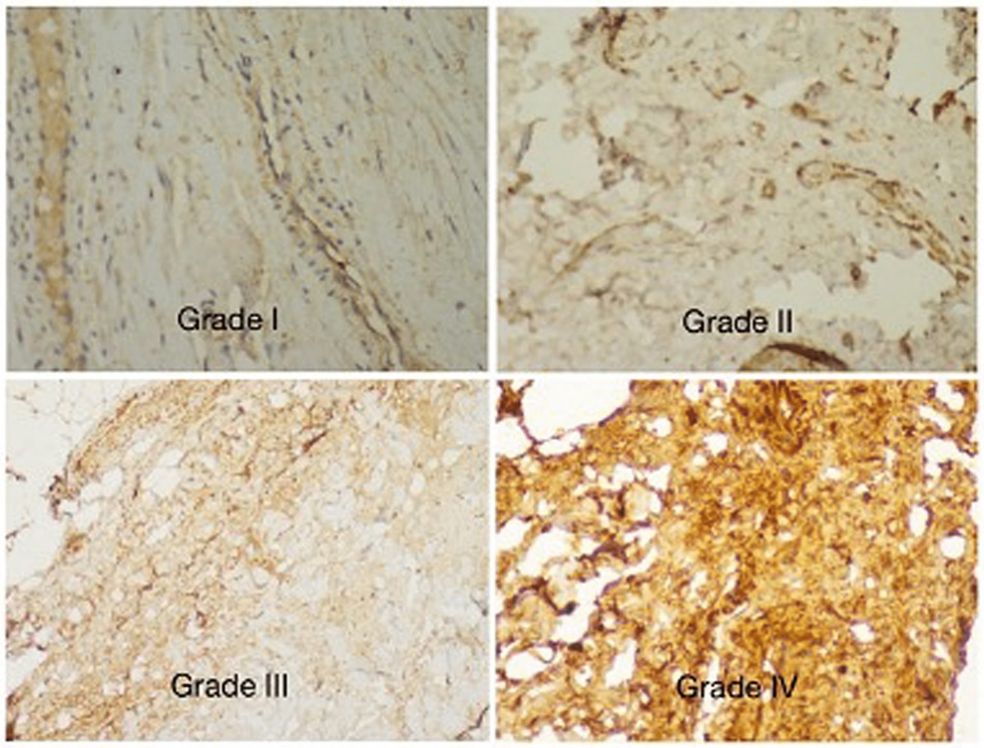
Grades of collagen staining in transversalis fascia

**Figure 3:**
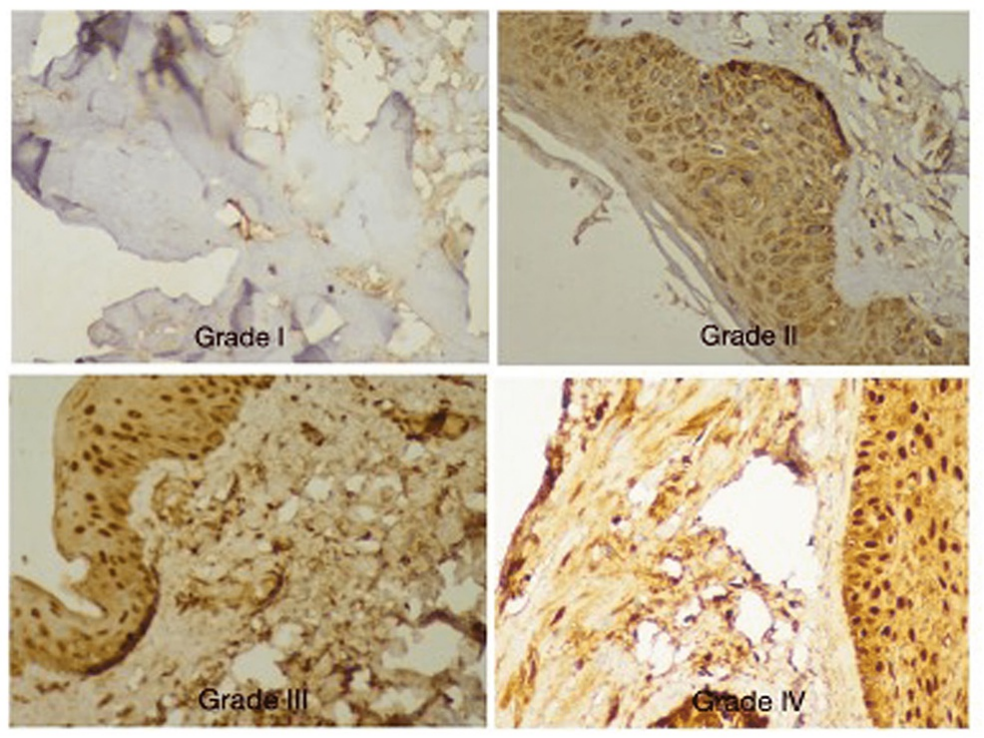
Grades of collagen staining in skin

The Masson’s trichrome stained slides were observed under polarized light. The collagen-filled areas were counted and in the areas without this element, the refringent areas were considered positive and assumed to be containing collagen (Figures 2, 3). The non-refringent areas were considered negative. The proportion between these two areas was noted in five random fields with 100 magnifications. These areas were further viewed under 400X magnification to distinguish between collagen I and III using immunohistochemical staining. 

Similar to the grading used in a study by Sun et al., we have graded the collagen staining in the skin and transversalis fascia depending on the percentage of positivity into four grades (Table 1) [12].

**Table 1:** Grading of collagen staining Grading was done similarly to Sun et al. in his study [12]

Grades	Percentage of positivity
Grade I	0% - 25%
Grade II	26% - 50%
Grade III	51% - 75%
Grade IV	76% - 100%

The Mann-Whitney U-test was used to analyze the results of collagen percentages, because of the non-parametric nature. Comparison between mean ages was done using the Students' t-test. In all tests, a P value of 0.05 or 5% was fixed as the level of significance. The ratio of collagen I to III was computed using the collected data. All data were analyzed with a statistical software package (SPSS for Windows, version 16.0, SPSS Inc., Chicago, IL). 

## Results

The mean $\pm$ SD age of patients with InH patients was 42.2 $\pm$ 14.5 years, while the mean age of controls was 41.6 $\pm$ 12.9 years. The difference between the mean ages was 0.6 years (p=0.8). 

**Figure 4:**
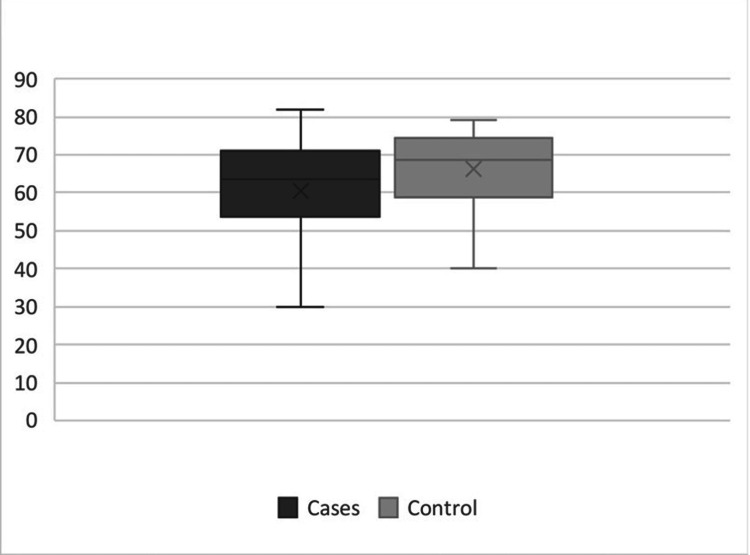
Collagen I percentages in the skin of patients and control (p=0.07, Mann-Whitney test)

**Figure 5:**
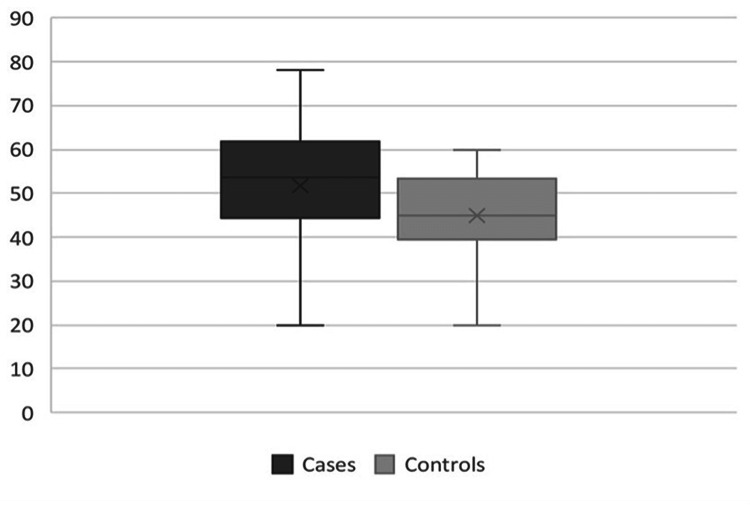
Collagen III percentages in the skin of patients and controls (p=0.04, Mann-Whitney test)

The mean percentage of collagen type I staining in the skin of InH patients was 60.4 $\pm$ 13.9%, while in the control group was 66.2 $\pm$ 10.5%. The difference between median percentages was 5%. the comparison between the groups was not significant (p=0.07) (Figure 4). The mean percentage of collagen type III staining in the skin of patients with hernia was 51.6 $\pm$ 15% and in controls was 44.9 $\pm$ 9.3%. The difference between median percentages was 8.5%. This comparison was noted to be significant (p=0.04) (Figure 5).

**Figure 6:**
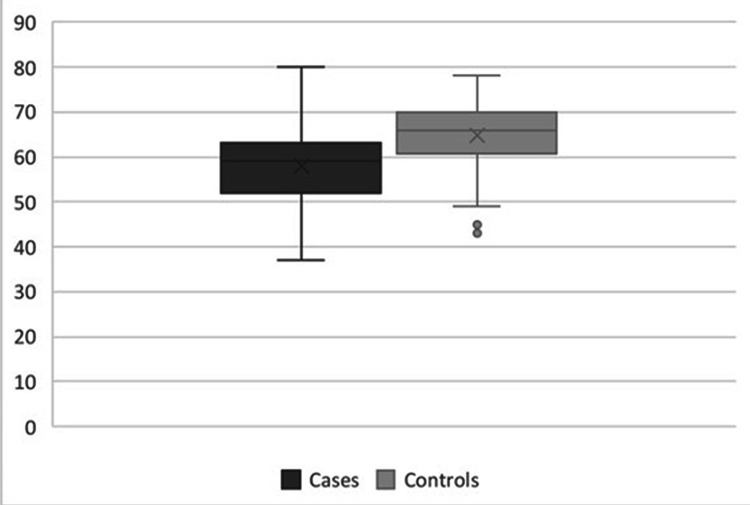
Collagen I percentages in transversalis fascia of patients and controls (p=0.007, Mann-Whitney test)

**Figure 7:**
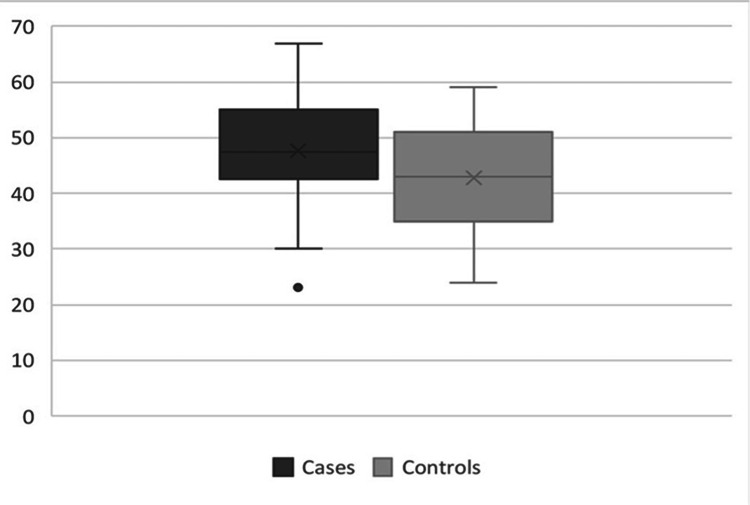
Collagen III percentages in transversalis fascia of patients and controls (p=0.08, Mann-Whitney test)

The mean percentage of collagen type I staining in transversalis fascia of InH patients was 58.0 $\pm$ 10.5%, while in the control group was 64.7 $\pm$ 8.24%. The difference between median percentages was 7%. the comparison between the groups was significant (p=0.007) (Figure 6). The mean percentage of collagen type III staining in transversalis fascia of patients with hernia was 47.3 $\pm$ 10.6% and in controls was 42.8 $\pm$ 9.1%. The difference between median percentages was 4.5%. This comparison was not significant (p=0.08) (Figure 7).

## Discussion

Medical science in the field of hernia has been progressing daily with the advent of newer modalities of management and techniques. It has embraced the idea of a weak abdominal wall biology. This “unified theory of hernia formation” was propounded by Robert Bendavid [13]. The fundamental mechanism of hernia formation has been found to be the loss of structural integrity at the musculotendinous layer [14].

Hence, choosing the right treatment means to account for the biochemical and metabolic mechanisms that are in play. Attempts have been to determine if there is a difference in the amount of collagen in the transversalis fascia of patients with hernia as compared to controls [15-17]. The importance of collagen has been stressed by many landmark studies [18]. Further studies have also propounded defective collagen synthesis in patients with hernia [3]. 

Although some studies have not found any significant change in collagen ratio even in patients with recurrent hernia, they were limited by small sample size and have been noted to compare the amount with the fascias of the same patient [19,20]. Other studies have been noted to use picosirius stains which have less sensitivity and specificity when compared with our technique of immunohistochemical staining [21]. While previous studies used cadavers as controls, in our study we have used live patients [22].

In our study, the InH patients were noted to have increased immature collagen (collagen III) in the skin and decreased mature collagen (collagen I) in the transversalis fascia as compared to the control group. This translated to a decreased ratio of collagen I to collagen III in both tissue samples as seen in other studies [15]. These findings strongly suggest the hypothesis of a weak transversalis fascia in patients with primary hernias. 

Studies have suggested skin as a representative sample for collagen [23]. This led us to compare not only cases and controls but to compare the two different tissues, skin, and transversalis fascia, separately among cases and controls. No significant difference was noted. So, the skin ratio of collagen was found to be representative of the transversalis fascia ratio in patients with InH. We recommend that a skin biopsy would suffice to identify a patient with unfavorable collagen distribution. 

Limitations of the study involved the sole use of immunohistochemical techniques. Ideally, we could have collaborated with other techniques such as western blotting. This could have delivered more objectivity. Due to financial constraints, we limited ourselves to collagen studies alone. 

## Conclusions

This study compared cases of inguinal hernia with age-matched live controls. Patients with inguinal hernias have been noted to have an increased quantity of immature collagen in the skin and decreased amount of mature collagen in the transversalis fascia. The skin sample of inguinal hernia patients was found to be representative of the transversalis fascia.
